# Supplementary material for: Definition of the Prognostic Role of MGMT Promoter Methylation Value by Pyrosequencing in Newly Diagnosed IDH Wild-Type Glioblastoma Patients Treated with Radiochemotherapy: A Large Multicenter Study
Source: Cancers (Basel). 2022 May 13;14(10):2425. doi: 10.3390/cancers14102425 (PMC9139569; doi:10.3390/cancers14102425)
Supplement: Supplementary file 1 [file cancers-14-02425-s001.zip › cancers-1627261-supplementary.pdf]

cancers

MDPI

Supplementary Material: Definition of the Prognostic Role of MGMT Promoter Methylation Value by Pyrosequencing in Newly Diagnosed IDH Wild-Type Glioblastoma Patients Treated with Radiochemotherapy: A Large Multicenter Study

Mario Caccese *, Matteo Simonelli, Veronica Villani, Simona Rizzato, Tamara Ius, Francesco Pasqualetti, Marco Russo, Roberta Rudà, Rosina Amoroso, Luisa Bellu, Roberta Bertorelle, Francesco Cavallin, Angelo Dipasquale, Mariantonia Carosi, Stefano Pizzolitto, Daniela Cesselli, Pasquale Persico, Beatrice Casini, Matteo Fassan, Vittorina Zagonel and Giuseppe Lombardi

Supplemental Material

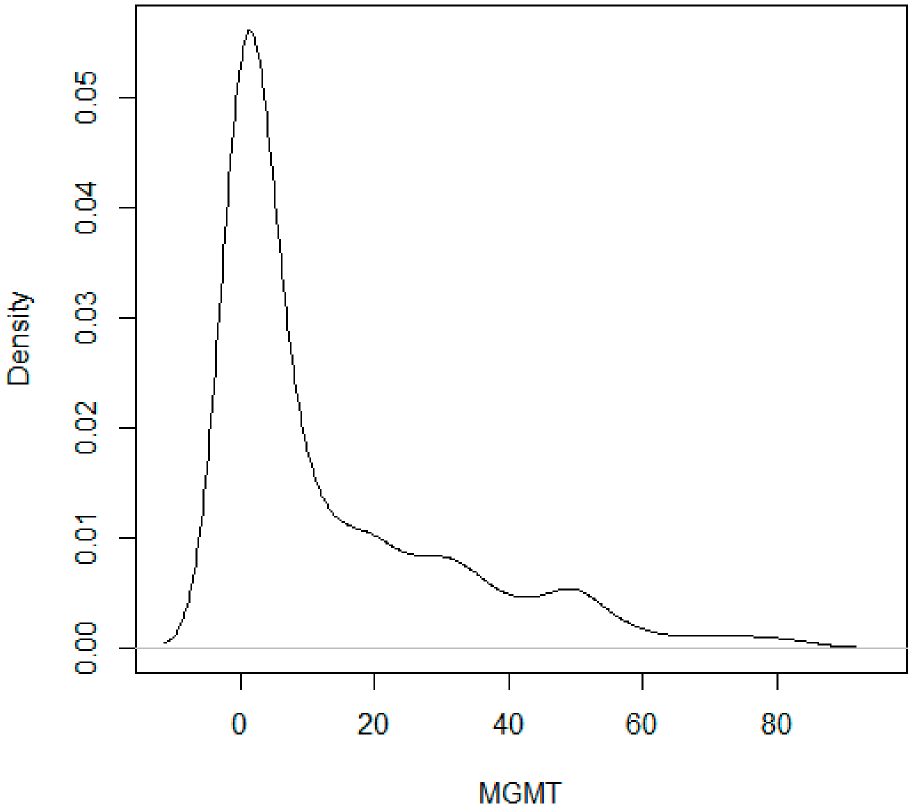

Figure S1: Distribution of MGMT promoter methylation in the study sample

Table S1. Characteristics of glioblastoma patients according to MGMT methylation status (<15% and ≥15%) by pyrosequencing.

Variable	MGMT methylation <15%	MGMT methylation ≥15%	*p*-value
n	401	190	-
Age at diagnosis, years [a]	60 (52–67)	61 (52–68)	0.81
Females	124 (30.9)	78 (41.1)	0.02
Males	277 (69.1)	112 (58.9)	
ECOG PS:			0.02
0	170 (42.4)	85 (44.7)	
1	173 (43.1)	93 (49.0)	

2	58 (14.5)	12 (6.3)	
Type of surgery: [b]			0.98
Radical	166 (41.6)	80 (42.1)	
Non-radical	233 (58.4)	110 (57.9)	
Second surgery	67 (17.4)	31 (16.8)	0.95

Data expressed as *n* (%) or a median (interquartile range). Data not available [b]2 and [c]20 patients. MGMT methylation was 0% in 94 males and 51 females.
